# Supplementary figures and images for: Conserved amino acid residues and gene expression patterns associated with the substrate preferences of the competing enzymes FLS and DFR
Source: PLoS One. 2024 Aug 28;19(8):e0305837. doi: 10.1371/journal.pone.0305837 (PMC11356453; doi:10.1371/journal.pone.0305837)

1.

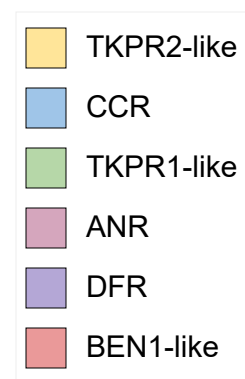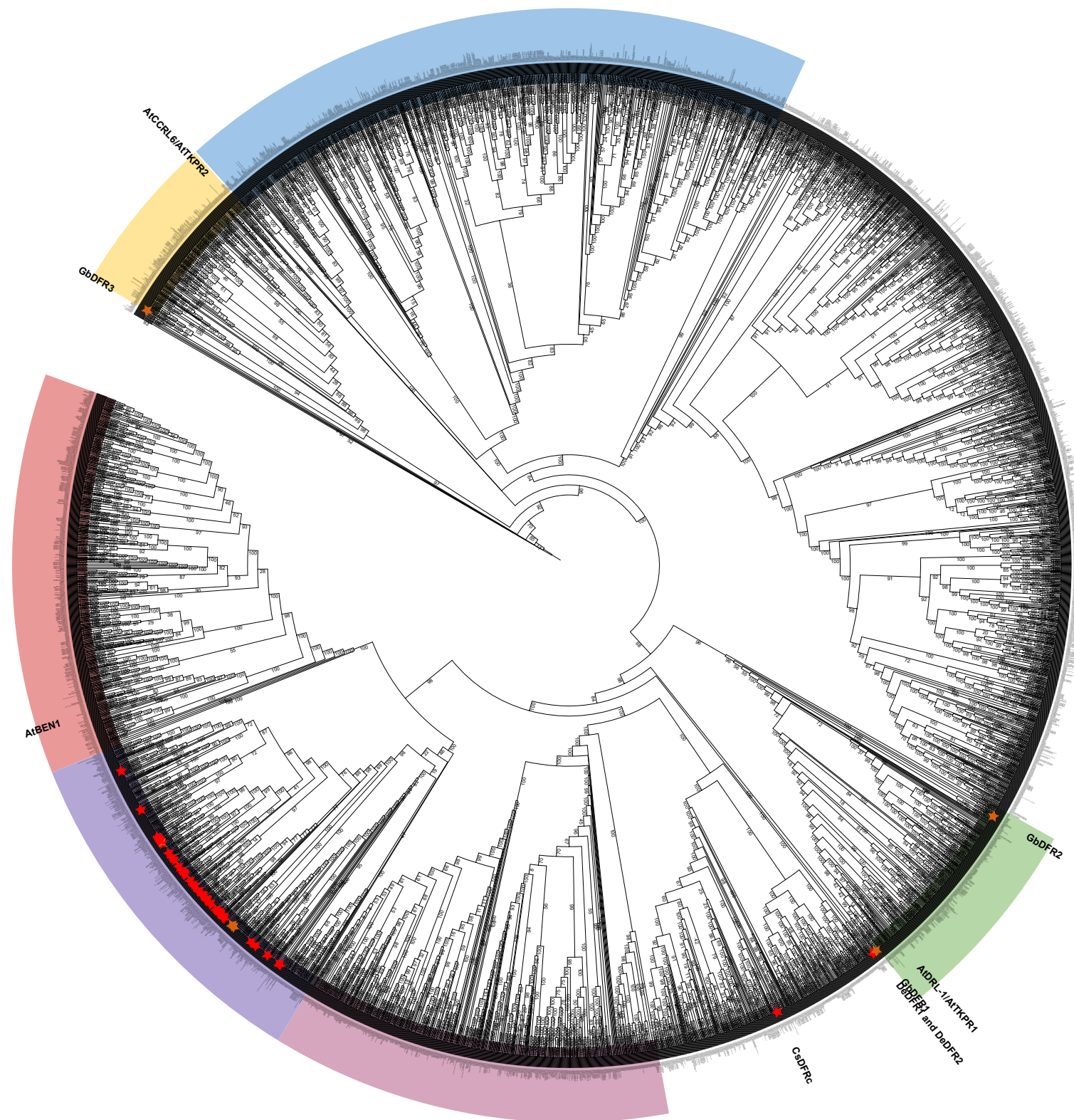

# 2a

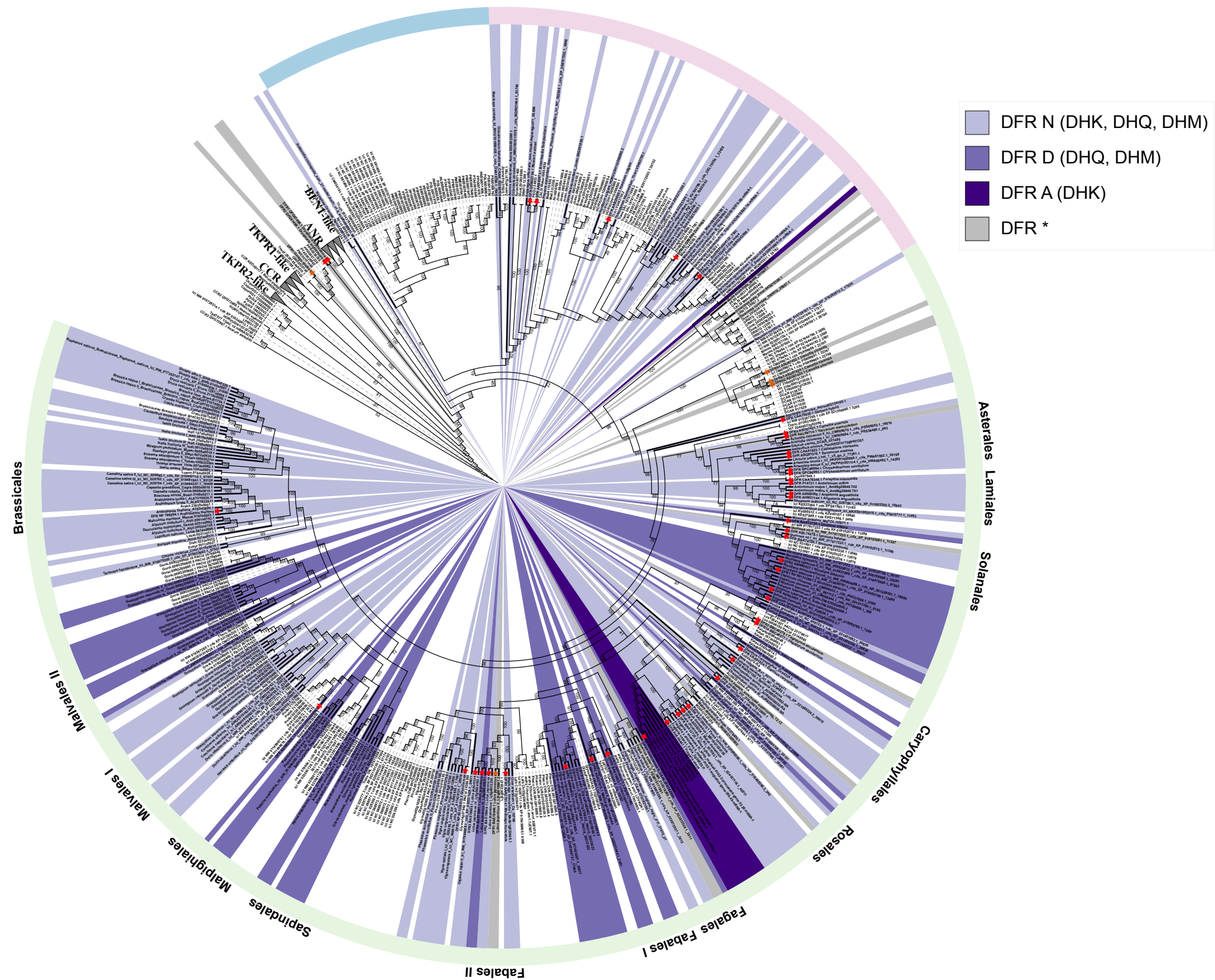

2b

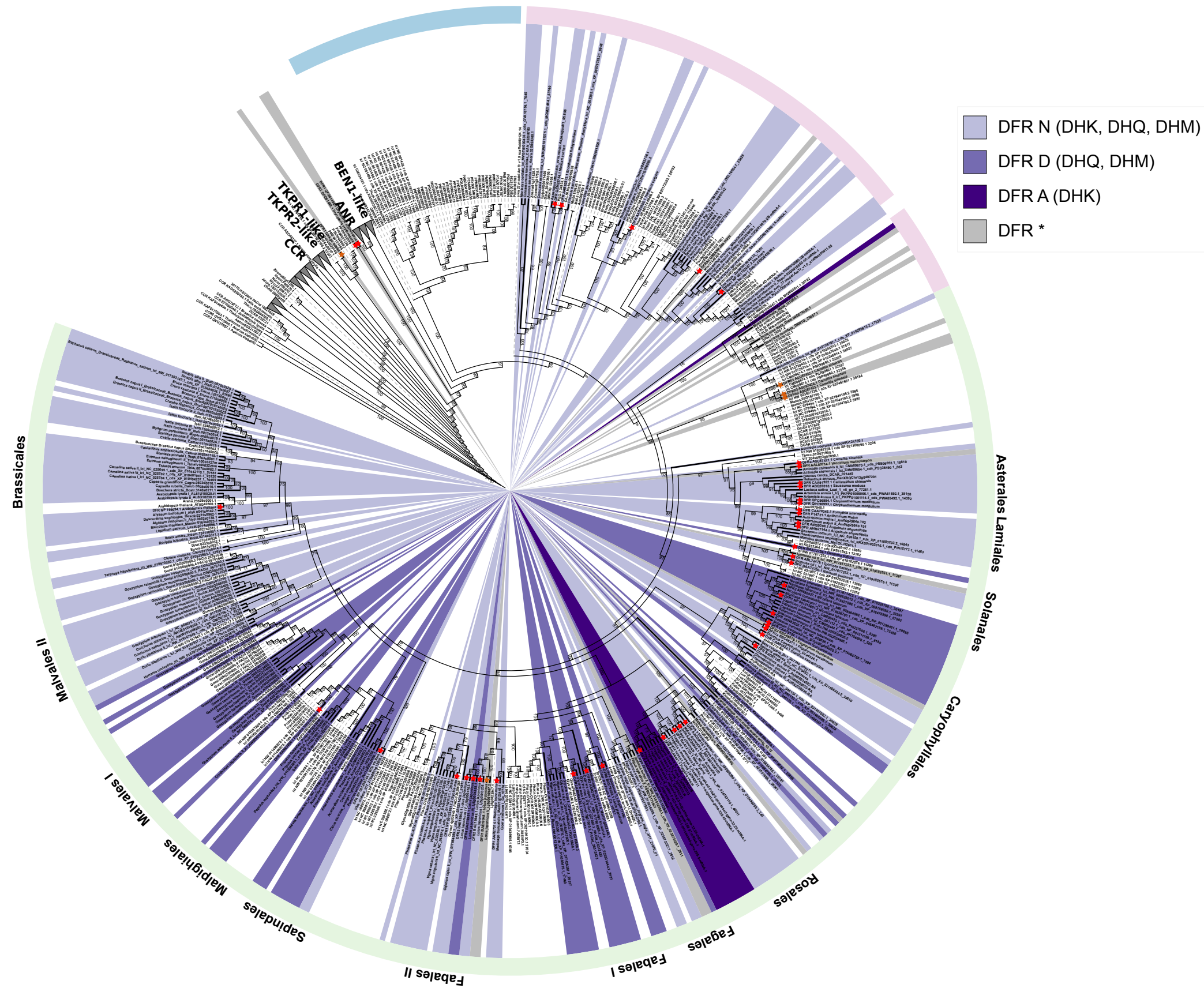

2c

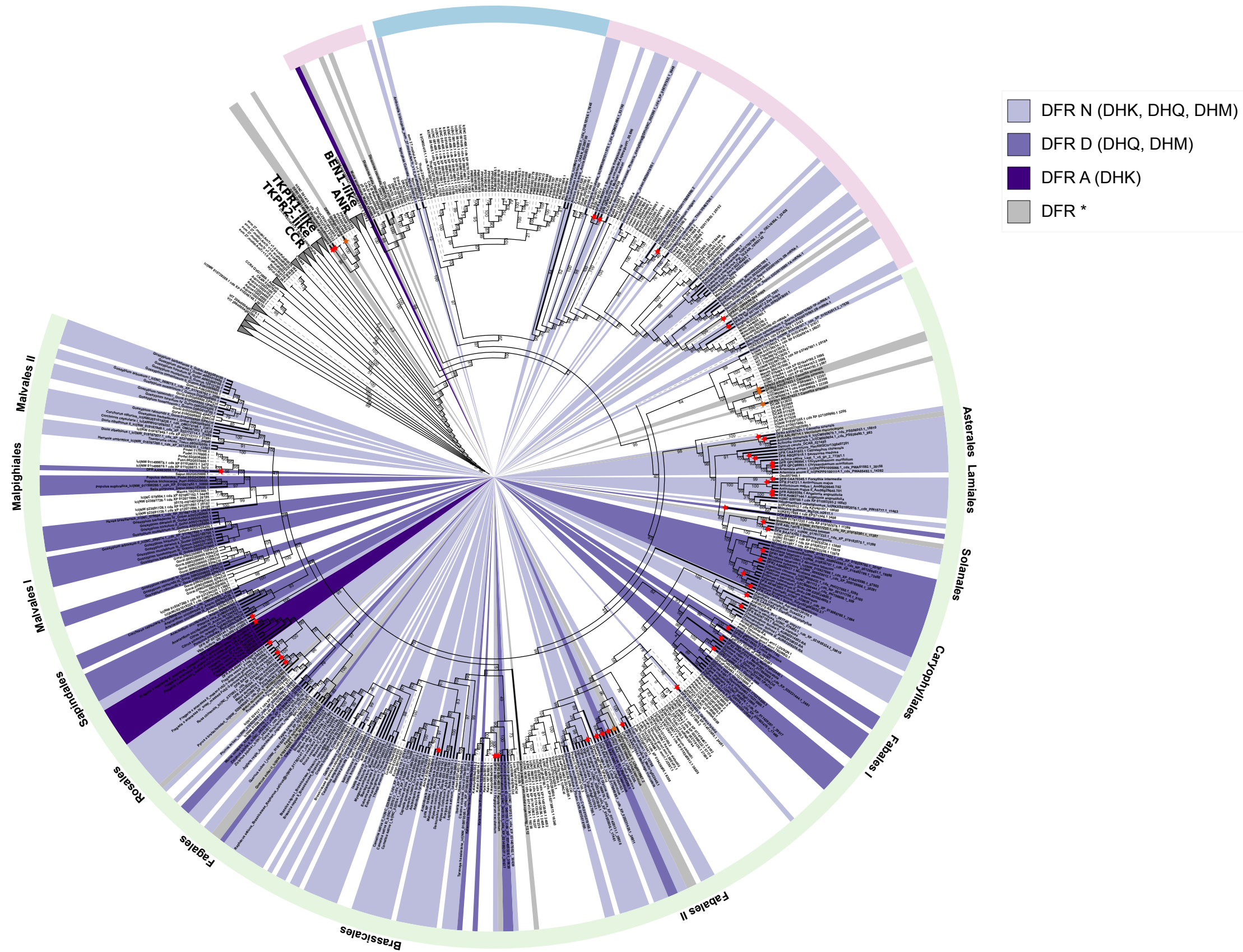

2d

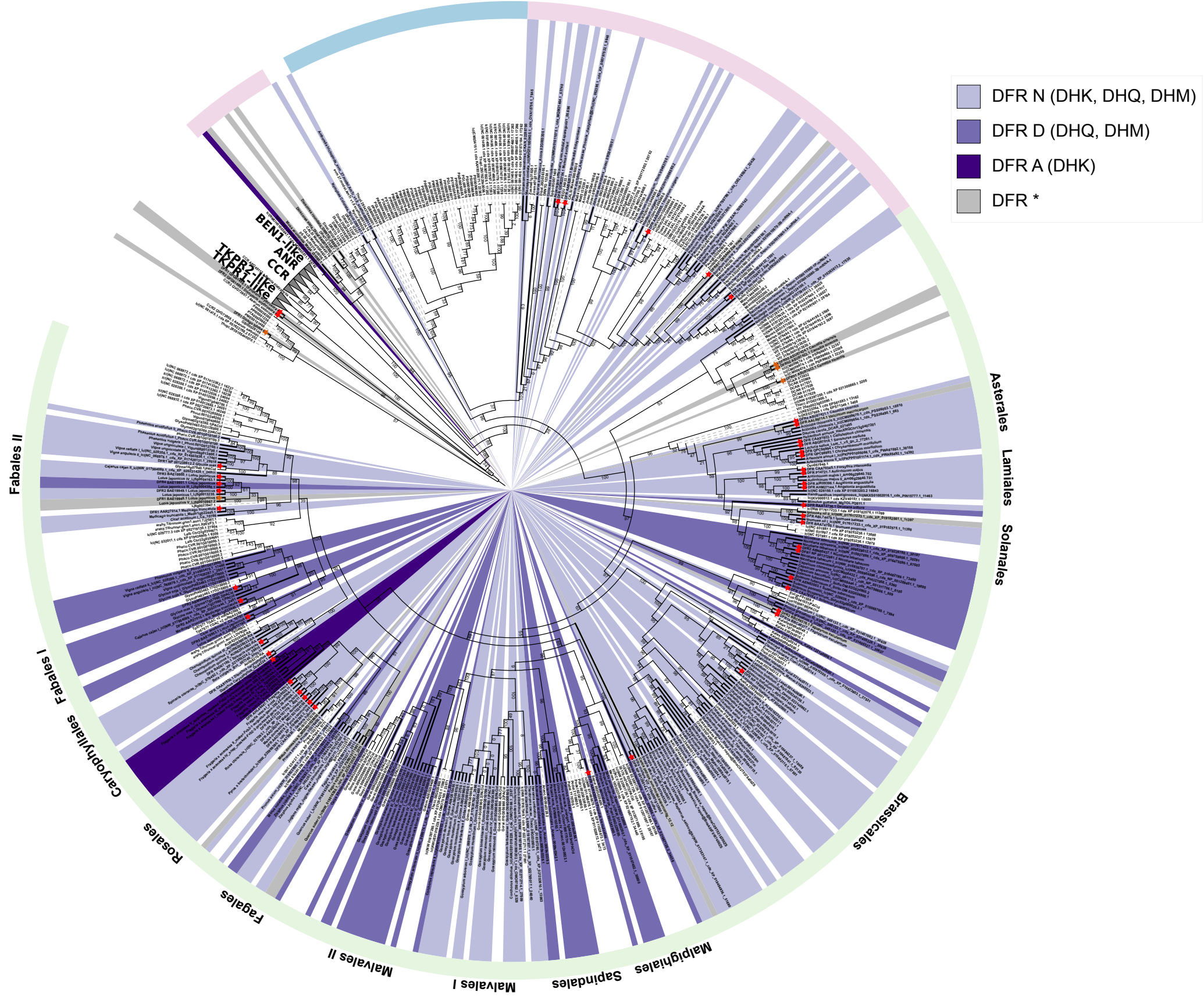

2e

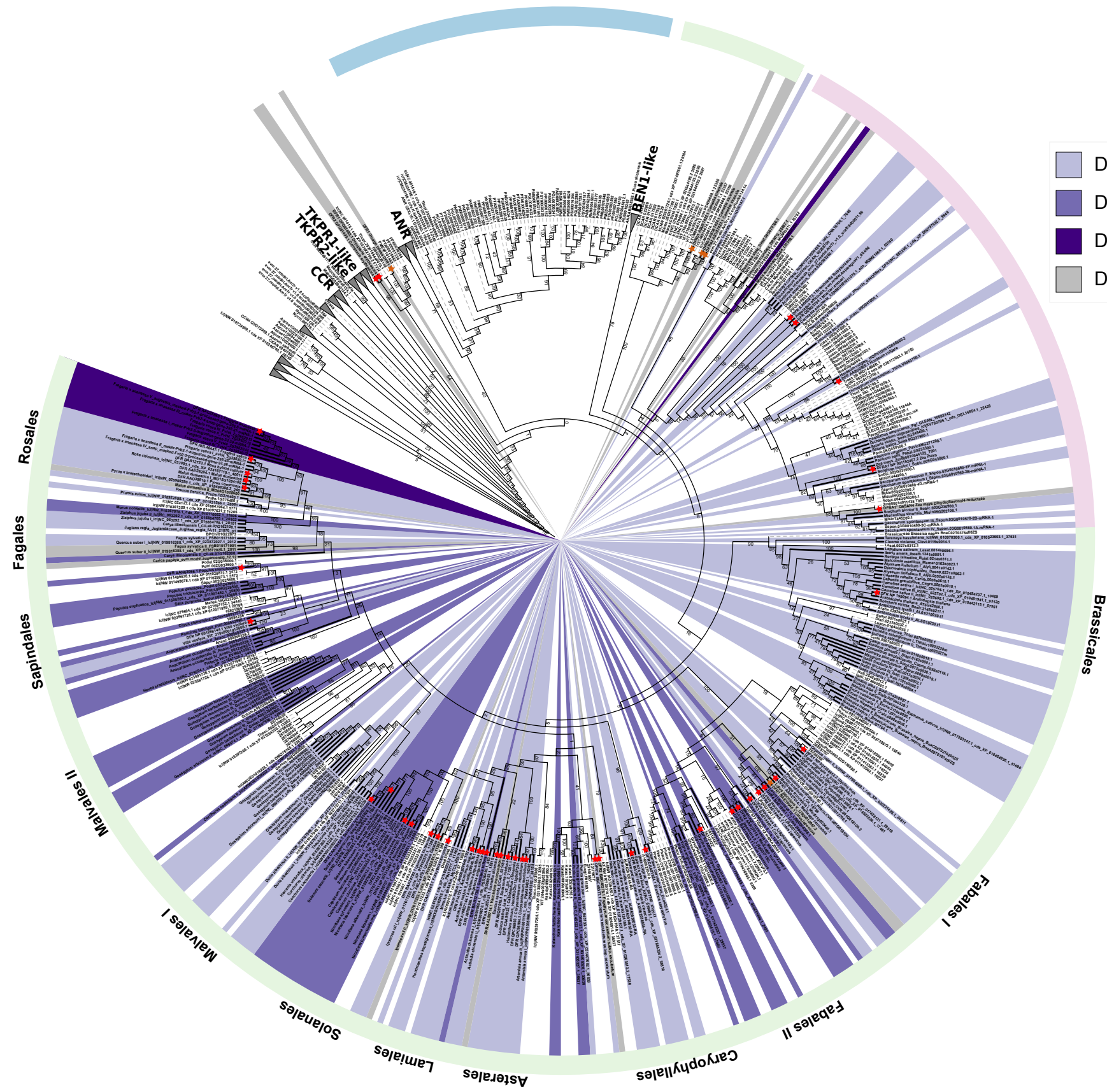

- DFR N (DHK, DHQ, DHM)
- DFR D (DHQ, DHM)
- DFR A (DHK)
- DFR \*

2f

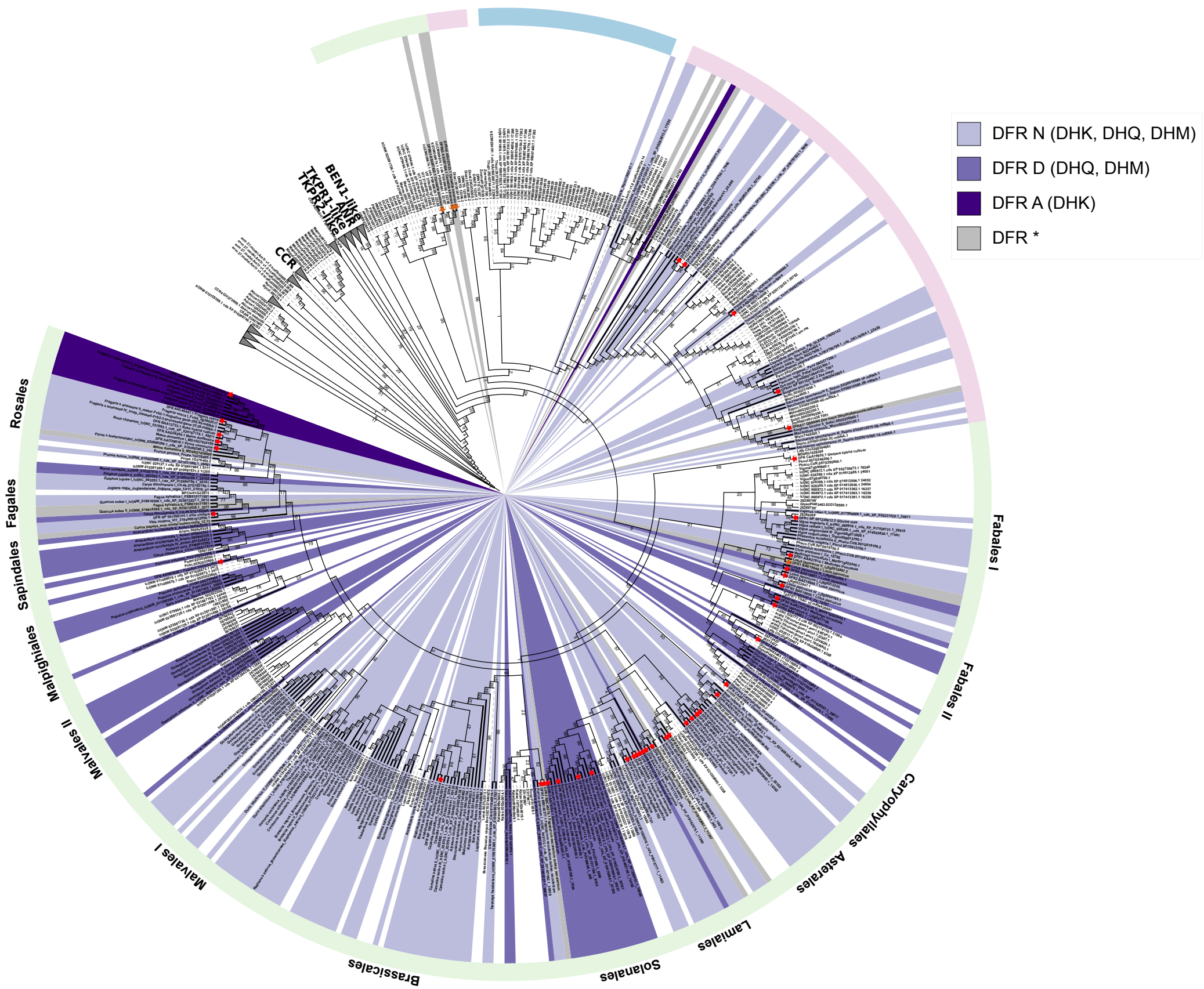

Supplement: S4 File — (1) DFR tree showing outgroups constructed by IQ-TREE based on an MAFFT alignment, (2a-f) DFR-specific trees with collapsed outgroups. Gymnosperm, monocot, and dicot species are denoted by light blue, light pink, and light green color stripes, respectively. Non-DFR sequences are represented by dashed gray branches while the functional DFRs are indicated by solid black branches. The color-coded scheme represents different substrate-preference-determining residues at position 133: Asparagine (light purple), Aspartic acid (periwinkle blue), Alanine (deep purple), and other amino acids (gray). The preferred substrate of the DFR type is written in brackets, DHK, dihydrokaempferol; DHQ, dihydroquercetin and DHM, dihydromyricetin. Distinct clusters of DFRs from major plant orders are labeled for reference. DFR sequences identified in previous studies are highlighted by an asterisk at the start of the terminal branch, with asterisks of functional DFR genes colored in red. (2a) Constructed by IQ-TREE based on a MAFFT alignment, (2b) constructed by IQ-TREE based on a Muscle5 alignment, (2c) constructed by FastTree2 based on a MAFFT alignment, (2d) constructed by FastTree2 based on a Muscle5 alignment, (2e) constructed by MEGA based on a MAFFT alignment, and (2f) constructed by MEGA based on a Muscle5 alignment. (PDF) [file pone.0305837.s004.pdf]

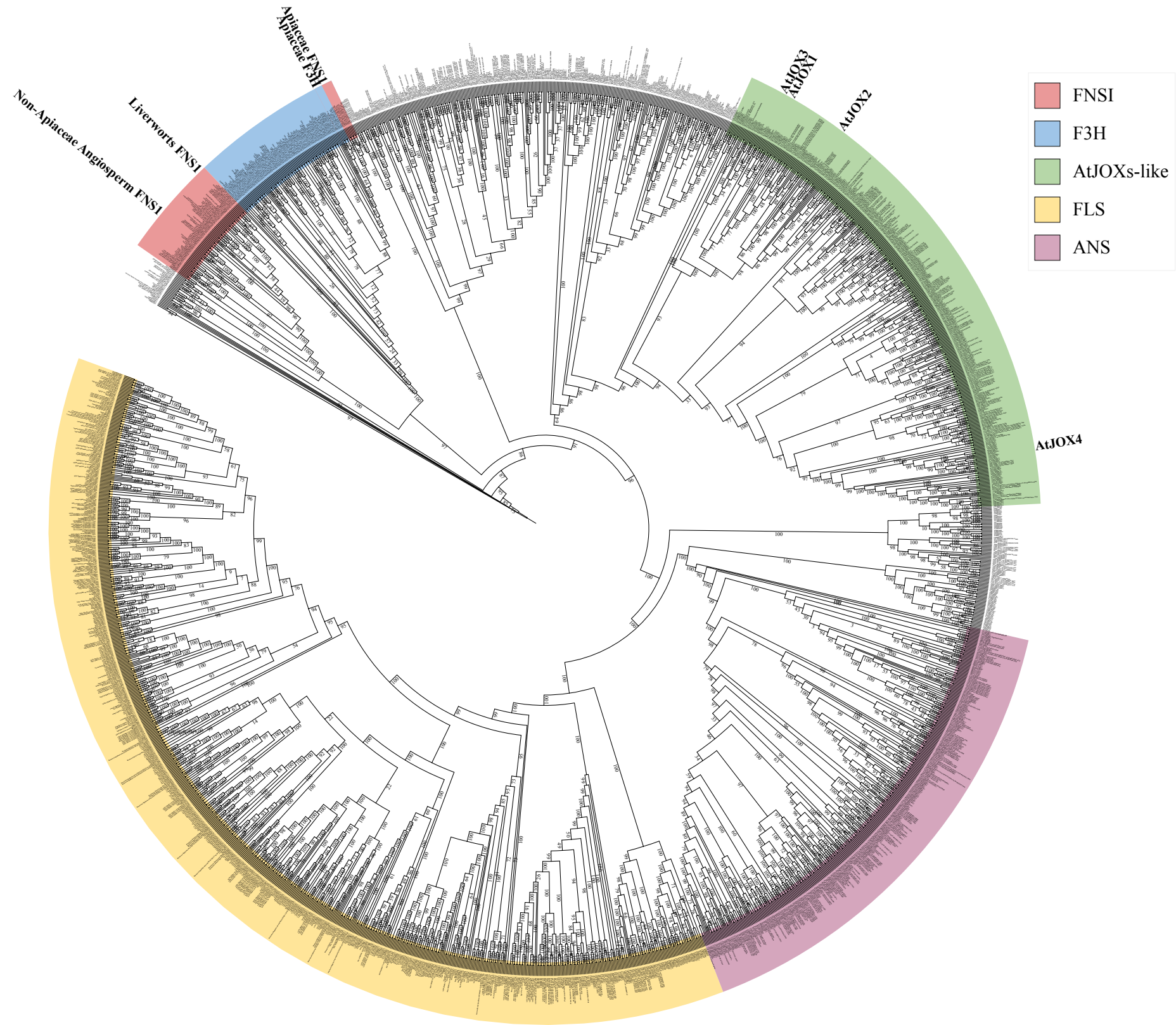

**2a**

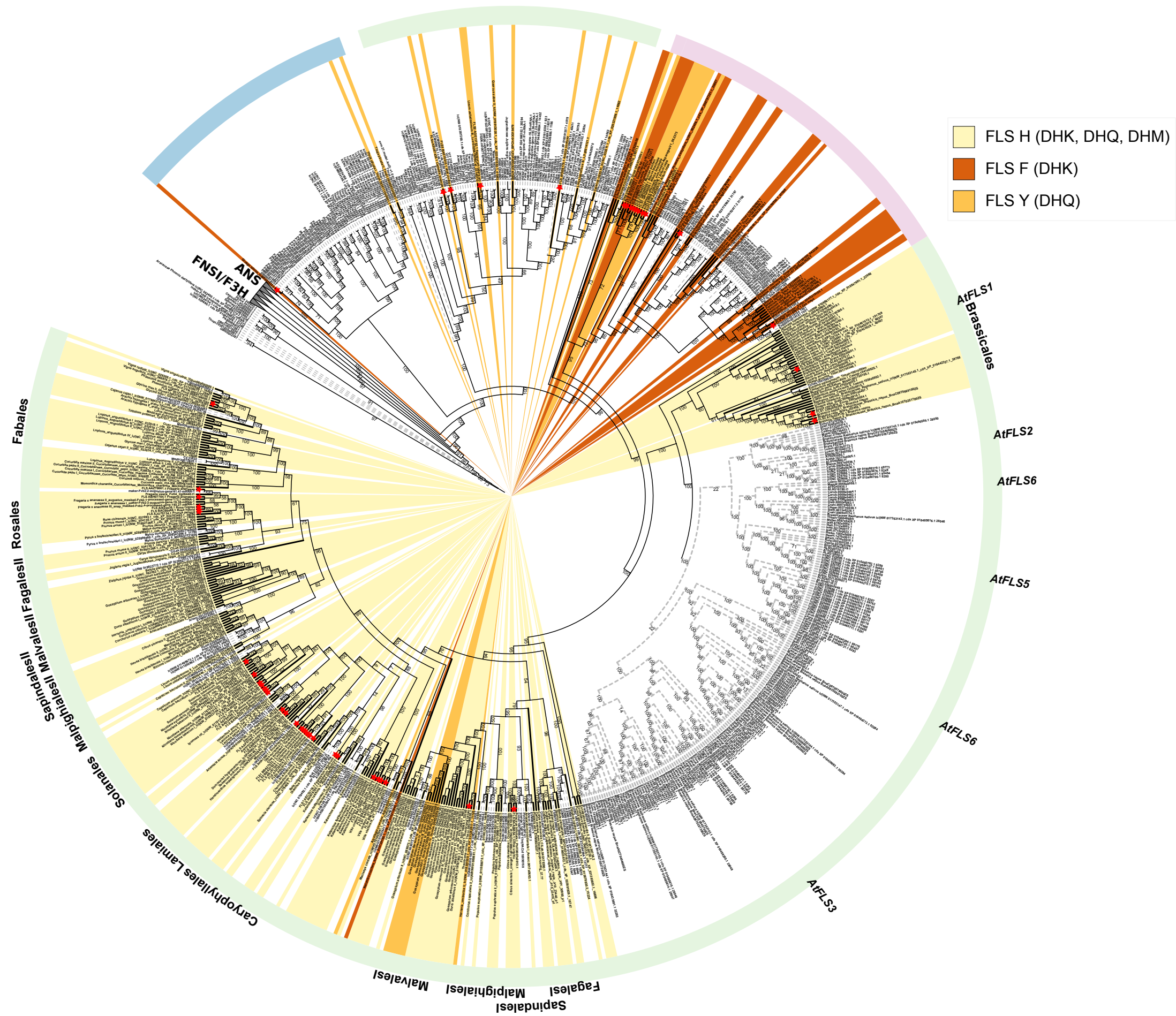

**2b**

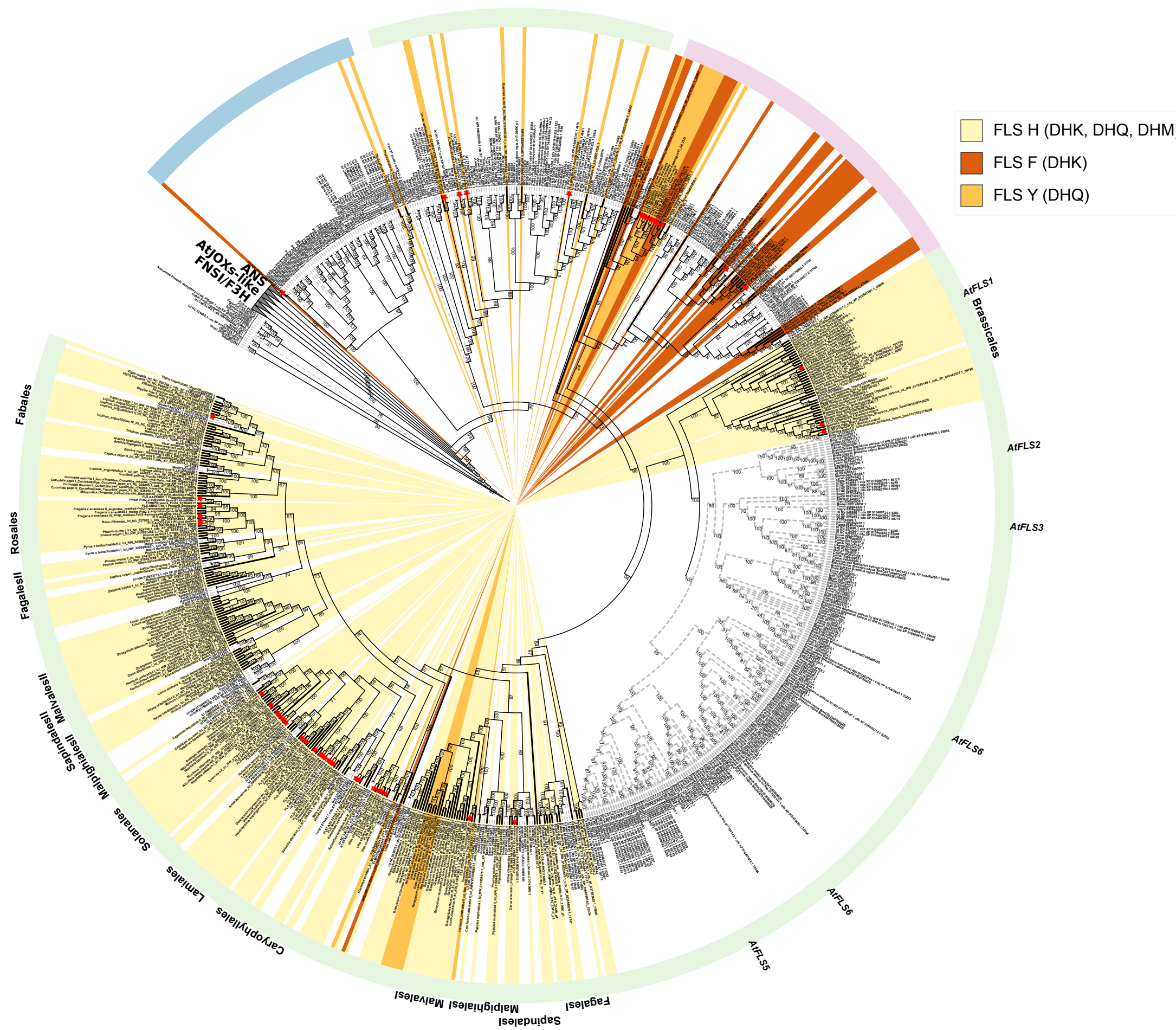

2c

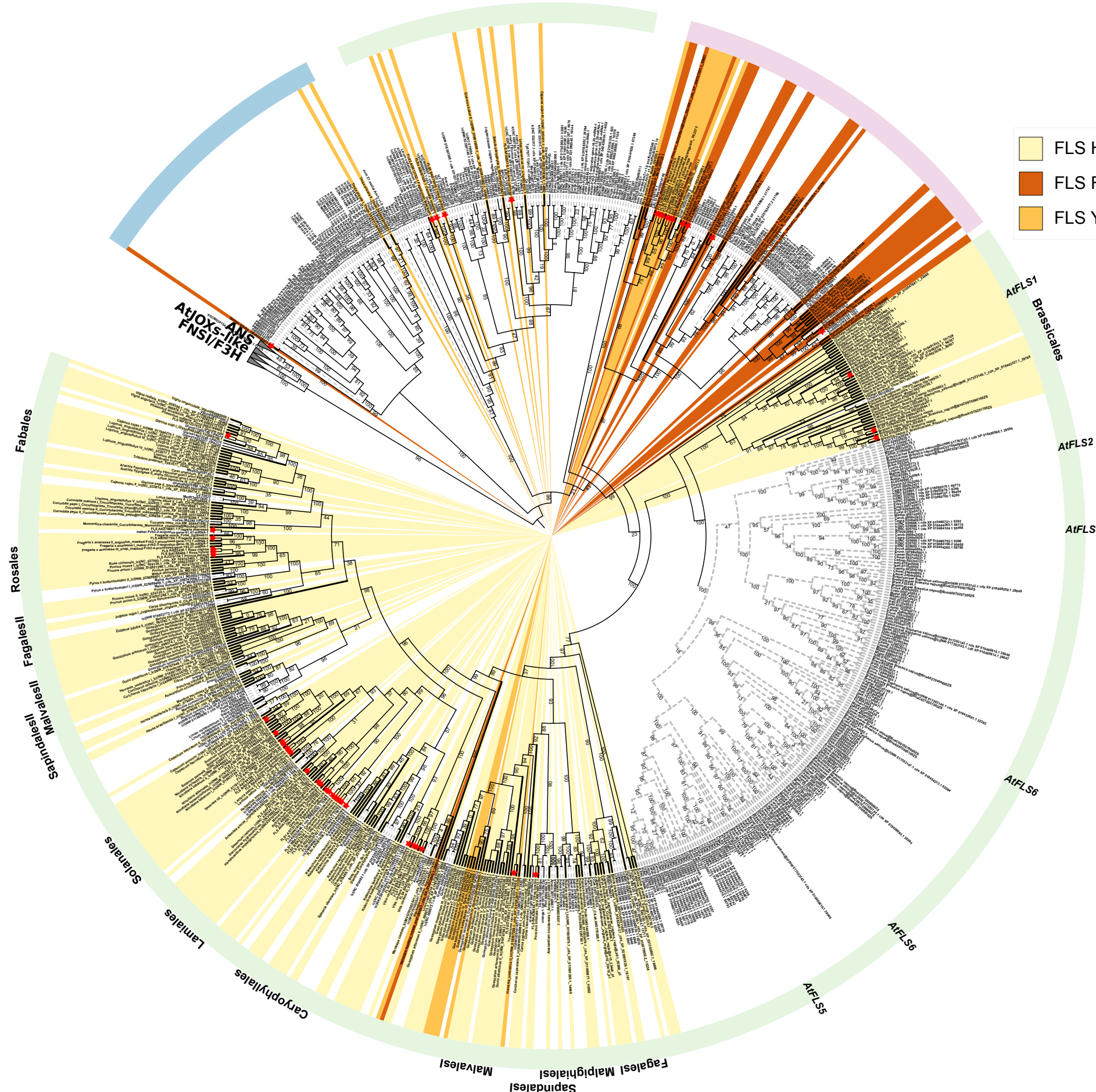

2d

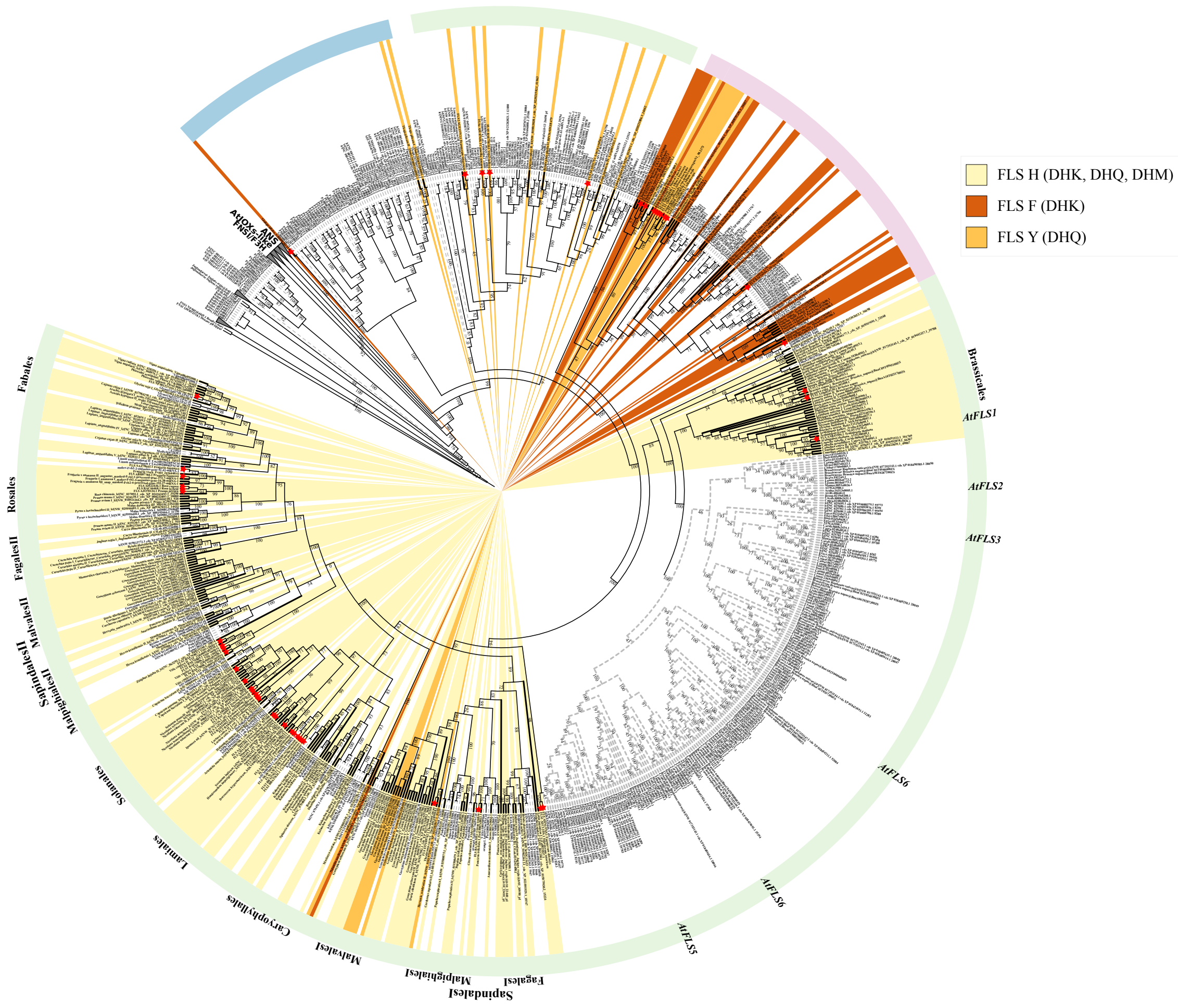

2e

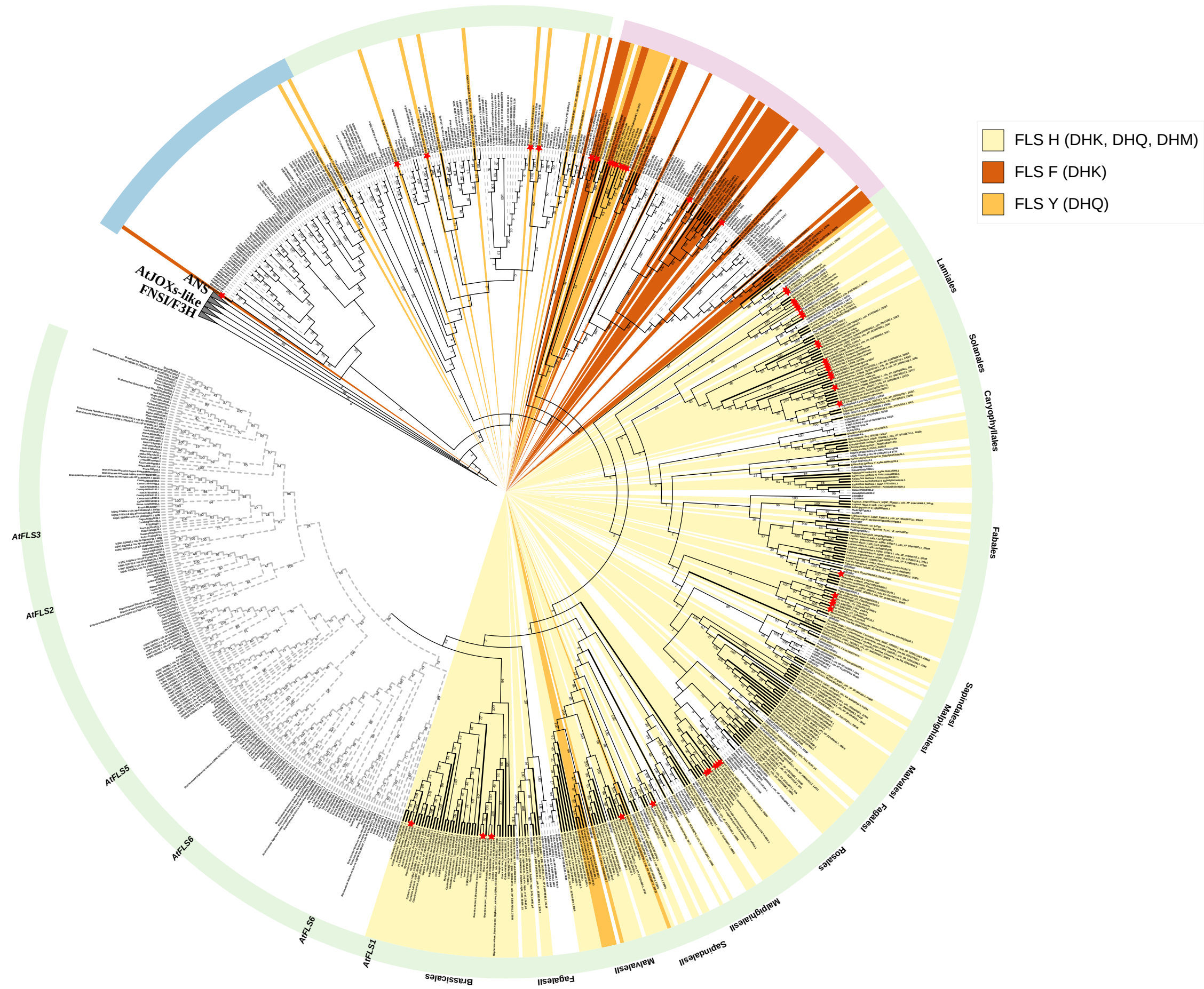

# 2f

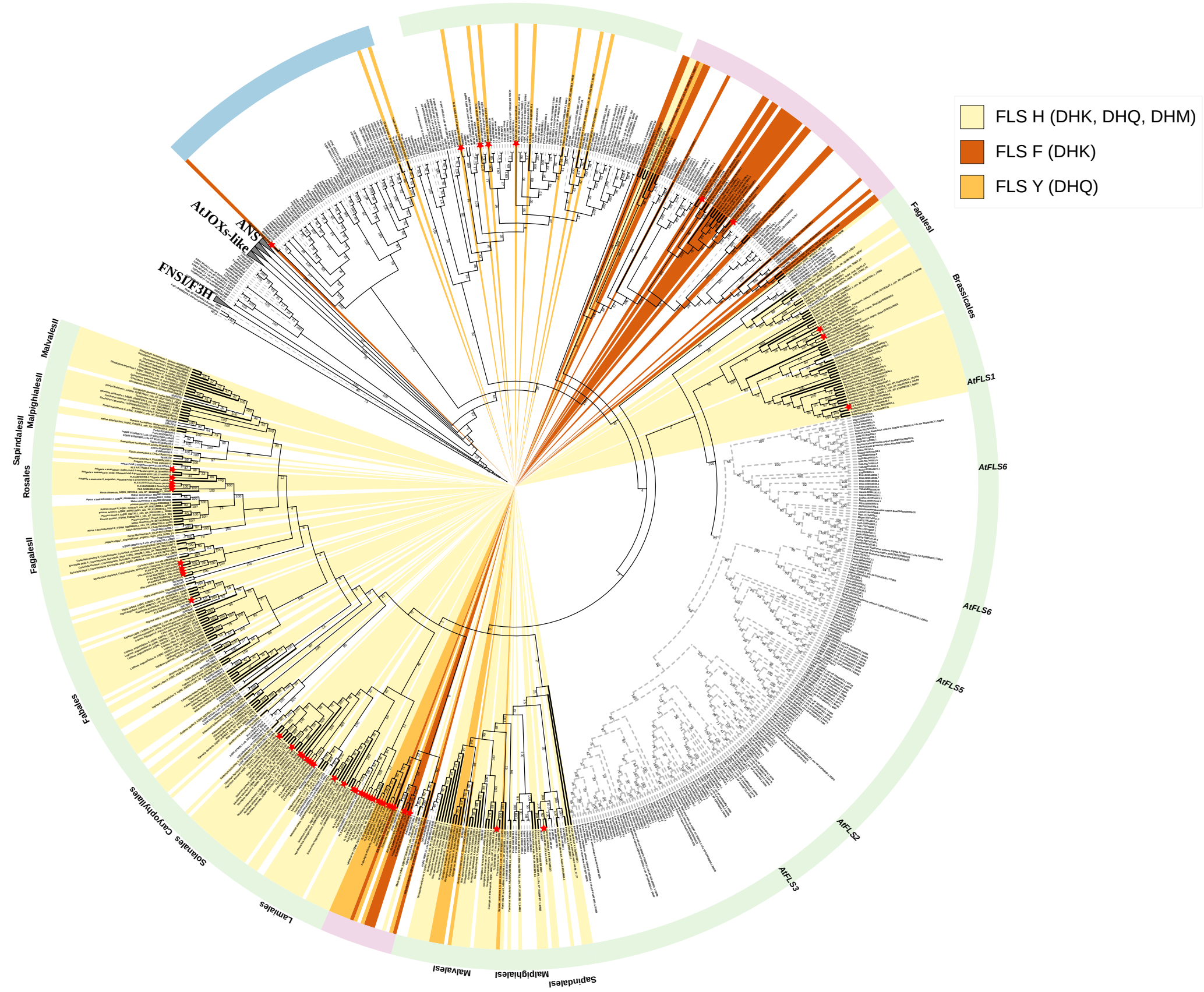

Supplement: S7 File — (1) FLS tree showing outgroups constructed by IQ-TREE based on an MAFFT alignment, (2a-f) FLS-specific trees with collapsed outgroups. Gymnosperm, monocot, and dicot species are denoted by light blue, light pink, and light green color stripes, respectively. Non-FLS sequences are represented by dashed gray branches while the functional FLSs are indicated by solid black branches. The background color highlights different presumably substrate-preference-determining amino acid residues at position 132: histidine (pale yellow), phenylalanine (dark orange), and tyrosine (dark golden). The hypothesized preferred substrate of the FLS type is written in brackets, DHK, dihydrokaempferol; DHQ, dihydroquercetin and DHM, dihydromyricetin. Distinct clusters of FLSs from major plant orders are labeled for reference. FLS sequences identified in previous studies are highlighted by an asterisk at the start of the terminal branch, with asterisks of functional FLS genes colored in red. (2a) Constructed by IQ-TREE based on a MAFFT alignment, (2b) constructed by IQ-TREE based on a Muscle5 alignment, (2c) constructed by FastTree2 based on a MAFFT alignment, (2d) constructed by FastTree2 based on a Muscle5 alignment, (2e) constructed by MEGA based on a MAFFT alignment, and (2f) constructed by MEGA based on a Muscle5 alignment. (PDF) [file pone.0305837.s007.pdf]

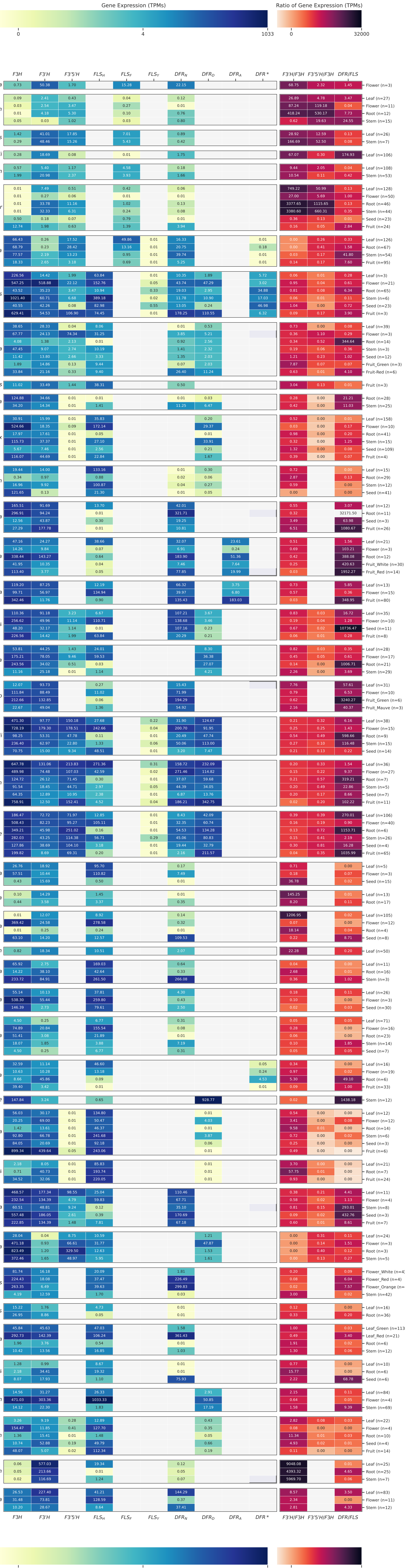

Supplement: S9 File — DFR* refers to DFR sequences where the residue 133 is not N, D, or A. In Musa acuminata C133 residue, Lotus japonicus S133, and Carica papaya S133. (PDF) [file pone.0305837.s009.pdf]
