# Supplementary material for: Conserved amino acid residues and gene expression patterns associated with the substrate preferences of the competing enzymes FLS and DFR
Source: PLoS One. 2024 Aug 28;19(8):e0305837. doi: 10.1371/journal.pone.0305837 (PMC11356453; doi:10.1371/journal.pone.0305837)
Supplement: S6 File — The codons and corresponding amino acids at position 133 are highlighted in red. The color-coded scheme represents different residues: Asparagine (light purple), Alanine (deep purple), and other amino acids (gray) at position 133. (PDF) [file pone.0305837.s006.pdf]

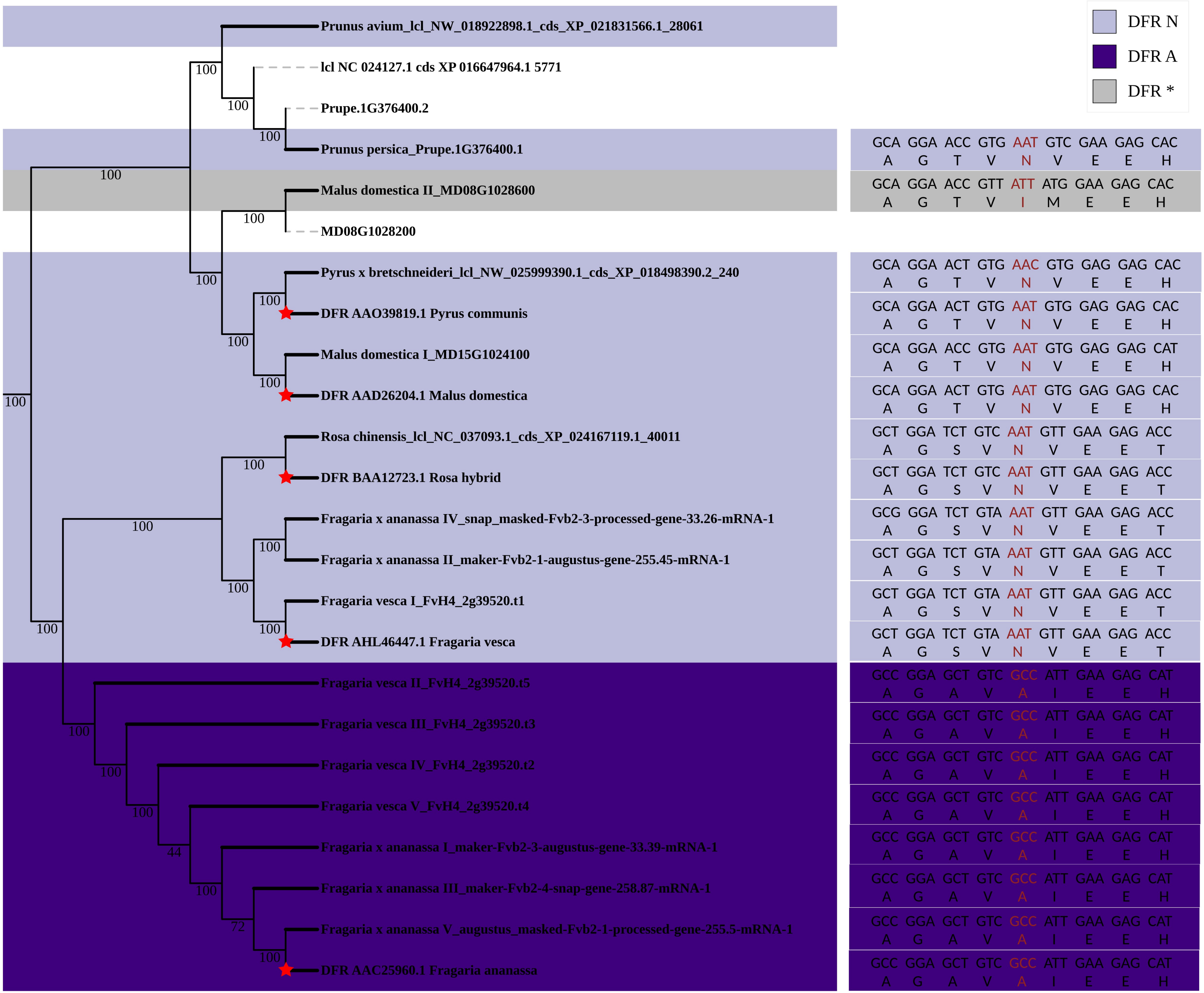
DFR N
DFR A
DFR *
Prunus avium_lcl_NW_018922898.1_cds_XP_021831566.1_28061
lcl NC 024127.1 cds XP 016647964.1 5771
Prupe.1G376400.2
Prunus persica_Prupe.1G376400.1
GCA GGA ACC GTG AAT GTC GAA GAG CAC
A G T V N V E E H
Malus domestica II_MD08G1028600
GCA GGA ACC GTT ATT ATG GAA GAG CAC
A G T V I M E E H
MD08G1028200
Pyrus x bretschneideri_lcl_NW_025999390.1_cds_XP_018498390.2_240
GCA GGA ACT GTG AAC GTG GAG GAG CAC
A G T V N V E E H
DFR AAO39819.1 Pyrus communis
GCA GGA ACT GTG AAT GTG GAG GAG CAC
A G T V N V E E H
Malus domestica I_MD15G1024100
GCA GGA ACC GTG AAT GTG GAG GAG CAT
A G T V N V E E H
DFR AAD26204.1 Malus domestica
GCA GGA ACT GTG AAT GTG GAG GAG CAC
A G T V N V E E H
Rosa chinensis_lcl_NC_037093.1_cds_XP_024167119.1_40011
GCT GGA TCT GTC AAT GTT GAA GAG ACC
A G S V N V E E T
DFR BAA12723.1 Rosa hybrid
GCT GGA TCT GTC AAT GTT GAA GAG ACC
A G S V N V E E T
Fragaria x ananassa IV_snap_masked-Fvb2-3-processed-gene-33.26-mRNA-1
GCG GGA TCT GTA AAT GTT GAA GAG ACC
A G S V N V E E T
Fragaria x ananassa II_maker-Fvb2-1-augustus-gene-255.45-mRNA-1
GCT GGA TCT GTA AAT GTT GAA GAG ACC
A G S V N V E E T
Fragaria vesca I_FvH4_2g39520.t1
GCT GGA TCT GTA AAT GTT GAA GAG ACC
A G S V N V E E T
DFR AHL46447.1 Fragaria vesca
GCT GGA TCT GTA AAT GTT GAA GAG ACC
A G S V N V E E T
Fragaria vesca II_FvH4_2g39520.t5
GCC GGA GCT GTC GCC ATT GAA GAG CAT
A G A V A I E E H
Fragaria vesca III_FvH4_2g39520.t3
GCC GGA GCT GTC GCC ATT GAA GAG CAT
A G A V A I E E H
Fragaria vesca IV_FvH4_2g39520.t2
GCC GGA GCT GTC GCC ATT GAA GAG CAT
A G A V A I E E H
Fragaria vesca V_FvH4_2g39520.t4
GCC GGA GCT GTC GCC ATT GAA GAG CAT
A G A V A I E E H
Fragaria x ananassa I_maker-Fvb2-3-augustus-gene-33.39-mRNA-1
GCC GGA GCT GTC GCC ATT GAA GAG CAT
A G A V A I E E H
Fragaria x ananassa III_maker-Fvb2-4-snap-gene-258.87-mRNA-1
GCC GGA GCT GTC GCC ATT GAA GAG CAT
A G A V A I E E H
Fragaria x ananassa V_augustus_masked-Fvb2-1-processed-gene-255.5-mRNA-1
GCC GGA GCT GTC GCC ATT GAA GAG CAT
A G A V A I E E H
DFR AAC25960.1 Fragaria ananassa
GCC GGA GCT GTC GCC ATT GAA GAG CAT
A G A V A I E E H
